# Is grandparental childcare socio-economically patterned? Evidence from the English longitudinal study of ageing

**DOI:** 10.1007/s10433-021-00675-x

**Published:** 2022-01-21

**Authors:** Giorgio Di Gessa, Karen Glaser, Paola Zaninotto

**Affiliations:** 1 Department of Epidemiology and Public Health, University College London, 1-19 Torrington Place, London, WC1E 7HB UK; 2 Department of Global Health and Social Medicine, King’s College London, London, UK

**Keywords:** Grandparents, Grandchild care, Frequency, Activities, Reasons, Socio-economic position, Education, Wealth

## Abstract

Grandparents play a vital role in providing childcare to families. Qualitative research and evidence from parents raise concerns that it is grandparents who are socio-economically disadvantaged who provide grandchild care more regularly, perform more intensive tasks, and care out of financial necessity. However, no European studies have investigated these issues at population level. This study is based on grandparents aged 50+ who looked after grandchildren. Data are from wave 8 of the nationally representative English Longitudinal Study of Ageing (2016/2017). We exploit newly collected information on frequency of grandchild care, activities, and reasons for care. Using multinomial regressions, we first examined the extent to which grandparents’ socio-economic characteristics (wealth and education) are associated with frequency of grandchild care. Second, using logistic regressions, we investigated whether wealth and education are associated with activities and reasons for grandchild care. Overall, grandparents from disadvantaged socio-economic backgrounds were more likely to provide more regular childcare. Similarly, grandparents in the lowest wealth quartile were more involved in hands-on activities (cooking, taking/collecting grandchildren to/from school), whereas highly educated grandparents were more likely to help grandchildren with homework. Finally, better-off grandparents were more likely to look after grandchildren to help parents and provide emotional support and less likely to report difficulty in refusing to provide care. Our findings show that grandparental childcare varies by socio-economic status with more intensive childcare activities falling disproportionately on those with fewer resources, and this may act to exacerbate existing socio-economic inequalities in later life.

**Supplementary Information:**

The online version contains supplementary material available at 10.1007/s10433-021-00675-x.

## Introduction

The role grandparents play in family life as providers of financial, emotional, and practical support has become increasingly important over the last decades (Hank et al. 2018; Herlofson and Hagestad 2012; Pulgaron et al. 2016). For instance, more than 50% of grandparents look after grandchildren in England (Di Gessa et al. 2020) and 20% of Italian grandchildren aged 0–13 are looked after by grandparents almost daily when their parents are at work (Zamberletti et al. 2018). However, more intensive childcare tasks may be falling disproportionately on grandparents with fewer resources, and this may exacerbate existing socio-economic inequalities in later life. For example, data from Europe and the USA suggest that grandparents living in households with their grandchildren as well as those who have ‘primary responsibility’ for raising a grandchild are more likely to be socio-economically disadvantaged compared to other grandparents (Fuller-Thomson and Minkler 2001; Glaser et al. 2018; Hayslip et al. 2019). Moreover, researchers have suggested that grandparents providing regular care undertake more demanding activities and help their grandchildren for financial reasons, highlighting further socio-economic disadvantage (McGarrigle et al. 2018; Peyton et al. 2001; Wheelock and Jones 2002). Although family experiences are important for understanding inequality, to the best of our knowledge, studies have largely ignored socio-economic differences in the kinship roles of older generations and more specifically in grandchild care (Brandt et al. 2021; McGarrigle et al. 2018; McLanahan and Percheski 2008).

Our study aims to fill this gap by examining whether, and to what extent, grandparents from different socio-economic backgrounds enact their roles differently and for different reasons. We assess associations between grandparents’ socio-economic status and both the frequency of childcare and the experience of grandparental childcare (activities undertaken and reasons for care). We use novel and recently collected data from the 2016/17 English Longitudinal Study of Ageing (ELSA). New and robust evidence is critical to understand if the role grandparents play in family life reflects socio-economic inequalities in later life.

### Theoretical links between socio-economic characteristics and grandparental childcare

We use the ‘Informal Care Model’ as a basis for explaining socio-economic differences in grandparental childcare. Although this model was originally designed to study the onset of informal care (defined as ‘the unpaid care provided to dependent persons by a person with whom they have a social relationship’), this framework can also be applied to explain individual variation in informal care provision (Broese van Groenou and De Boer 2016), including grandparental childcare, a form of family support. The Informal Care Model posits that becoming a carer is not a random process. Three basic elements need to be considered to fully understand why some individuals assume caring roles: first, the care receiver’s need for care; second, the individual dispositional factors; and third, external conditions that can facilitate or restrict the provision of care. Assuming that the care recipient’s needs are the most important driver for the onset of informal care, the decision to then provide care is driven by an individual’s ability and willingness which are shaped by multiple and often inter-related factors. For example, an individual’s disposition to provide care is formed by attitudes and beliefs that are in turned shaped by a range of socio-cultural norms at both the individual and country levels, including gender-related expectations around care, attitudes towards the norm that family should be responsible for care, the extent to which care is provided out of affection, altruistic behaviour, and reciprocity, or the degree to which someone feels ‘obliged’ or ‘expected’ to provide care from societal, cultural, or family pressures (Al-Janabi et al. 2018; Greenwood and Smith 2019). In addition to these factors, competence, time, distance, or financial resources can also influence the individual disposition of carers. For instance, poor health of the carer and geographical distance between carers and care recipients are likely to limit the provision of care (Baldassar et al. 2007; Bauer and Sousa-Poza 2015; Szinovacz and Davey 2008). Individual decisions to provide care may also reflect more rational ‘cost/benefit’ calculations linked to factors such as loss of income, cost of formal care, or health and well-being consequences derived from the provision of care (Brouwer et al. 2005). For instance, people in full-time employment and higher earners are less likely to take on intensive caring responsibilities (Carmichael et al. 2010). Finally, the Informal Care Model posits that contextual factors may also facilitate or hinder the provision of care, with family size and composition, social networks, the availability of formal support via the community, as well as macro-level economic and societal policies influencing individuals’ care uptake.

In line with these arguments and following the applicability of the Informal Care Model, in this study we use this framework to investigate differences in grandparental childcare by socio-economic status. In particular, if competence, time, distance, and financial resources can all impact care (including grandparental childcare), we expect grandparents in lower socio-economic groups to provide more regular grandchild care than those who are better off for several reasons. These include individuals in lower socio-economic groups having fewer time constraints resulting from engagement in voluntary activities (Niebuur et al. 2018); being more likely to maintain a traditional extended family structure characterised by geographic proximity (or coresidence) and frequent in-person contacts across generations (Coimbra et al. 2013); and having fewer resources to access private care (Broese van Groenou et al. 2006; Schram et al. 2019). Therefore, we expect that poorer families are overall more likely to respond to the needs of the care recipients by providing more regular care and by helping with more hands-on and time-intensive tasks, in comparison with those who are better off because the latter have more resources to access, purchase, and use alternative forms of care, help, and support from the market (de Zwart et al. 2017; Saito et al. 2018). As for education, we expect more educated grandparents, compared to those with lower educational levels, to be less involved in grandchild care and more selective of what activities they undertake with their grandchildren as they have more active lives outside the family (e.g. through work or volunteering) (Arpino and Bordone 2017). We also expect them to have greater agency in choosing their level of involvement with grandchildren (McGarrigle et al. 2018; Mueller et al. 2002). Higher education is generally associated with greater need for self-development and involvement in more diverse leisure activities, higher levels of personal independence, and greater agency to negotiate caring roles within the family (Bye and Pushkar 2009; Conlon et al. 2014; Longest et al. 2013; Stalker 2011). Education has also been associated with parenting practices and child-rearing, with highly educated individuals more likely to afford and value toys, books, or activities that foster curiosity and cognition (Bornstein and Putnick 2012) and similar mechanisms may be observed among grandparents providing grandchild care. Yet, to date, few studies have addressed socio-economic disparities in grandparental childcare.

### Empirical findings on socio-economic characteristics and grandparental childcare

As described below, most of the evidence on the associations between socio-economic characteristics and grandparental childcare is based on families receiving childcare from grandparents (rather than on the providers of care themselves), on qualitative studies, or on socio-economic differences between grandparents who look after grandchildren and those who do not, overlooking differences in the characteristics of the grandchild care providers (Airey et al. 2020; Arpino et al. 2014; Glaser et al. 2013; Hank and Buber 2009; Huskinson et al. 2016; Kanji 2018; McGarrigle et al. 2018). Moreover, no evidence exists at a population level in Europe on whether grandparents’ socio-economic characteristics are associated with activities undertaken and reasons for care (Hank et al. 2018).

Evidence based on parental/family circumstances broadly suggests that, although families across all socio-demographic groups rely on grandparents, there is a greater propensity for those in lower socio-economic groups to use informal childcare (Huskinson et al. 2016) (Speight et al. 2009). For example, in Italy, Arpino et al. (2014) found that it is mostly socio-economically disadvantaged mothers who rely on grandparents as primary sources of childcare. Qualitative studies on grandparents also suggest that family’s socio-economic circumstances influence the amount and nature of childcare provided. Grandparents provided more frequent grandchild care in families where mothers were in low-paid or insecure jobs (Airey et al. 2020), whereas those in higher socio-economic groups were less likely to provide intensive grandchild care as they preferred and were ‘busy with’ social and leisure activities, and their children were better able to pay for formal child care (McGarrigle et al. 2018). The scarce body of quantitative evidence on the association between grandparents’ socio-economic characteristics and their involvement in childcare suggests that more economically disadvantaged grandparents may not have the financial resources to help their children with formal childcare and may end up giving more practical help by looking after grandchildren (Gray 2005). Research based on the Survey of Health, Ageing, and Retirement in Europe suggests that grandparents with higher levels of education are less likely to look after grandchildren intensively (Di Gessa et al. 2016; Glaser et al. 2013; Igel and Szyklik 2011). However, a recent study based on Italian grandparents found few education and wealth differences among grandparents providing childcare, with those in the higher rather than lower status groups being more likely to take on such family responsibilities (Zamberletti et al. 2018).

There is also some evidence to suggest that activities undertaken and reasons for grandchild care may vary by socio-economic factors. For instance, evidence from the USA suggests complex relationships between education and grandparental childcare, with those in the less educated groups more likely to worry about their grandchildren’s future, to view grandchildren as important for family continuity, as well as to report more contacts and feel closer to their grandchildren (King and Elder 1998). Grandparents with higher levels of education were more engaged in ‘activities they are more likely to be good at by virtue of having more education’ such as giving advice and participating in activities including going to a museum or attending events (King and Elder 1998:469). Similarly, differences across socio-economic groups in grandparental childcare are likely to reflect diverse expectations and reasons for looking after grandchildren. For instance, McGarrigle et al. (2018) found that grandparents in higher socio-economic groups were more likely to state that their involvement with their grandchildren arose from choice and a desire to have a positive impact on their development, whereas grandparents in lower socio-economic groups were more constrained and were often less able to ‘say no’.

### Aim and research questions

Our work aims to fill an important gap by examining whether and to what extent grandparents’ socio-economic characteristics are associated with the frequency of grandchild care provision, activities, and reasons for care. Based on the Informal Care Model framework and previous empirical research providing some insight into such associations, we expect to find that grandparents with fewer financial resources provide more frequent grandchild care, undertake more hands-on and time-intensive tasks, and care mostly due to financial necessity in the adult child’s family and for involuntary reasons. Similarly, as education mostly influences how grandparents think about and enact their roles, grandparents with higher levels of education may perceive grandparental childcare duties as a barrier to other (particularly leisure) activities and therefore may be more selective of their engagement in grandchild care and less likely to feel obliged to look after grandchildren. To our knowledge, no previous European studies have investigated these issues at a population level. Taken together, our study—exploiting newly collected data on grandparenting experiences in England (see below)—aims to better understand socio-economic differences in grandparental childcare.

## Methods

### Study population

We based our study on ELSA, an ongoing multidisciplinary longitudinal biennial survey of individuals aged 50 and over (Steptoe et al. 2012). In the first wave collected in 2002/03, around 12,000 respondents were recruited to provide a representative sample of the population aged 50 and over living in private households in England (household response rate was 70%). More details of the survey’s sampling frame, methodology, and questionnaires have been reported elsewhere (https://www.elsa-project.ac.uk). Data were drawn from the eighth wave of the study, collected in 2016/17, based on 8,445 individual interviews. Wave 8 was the first wave of ELSA which introduced a new module on grandparenting, hence the focus on this wave of the study. Analyses were restricted to respondents who had at least one grandchild under the age of 16 and who provided childcare, resulting in a sample of 2,769 grandparents.

### Outcome variables

#### Frequency of grandchild care

All grandparents were asked whether they looked after any grandchildren without their parents being present during the 12 months prior to the interview. Those who reported looking after grandchildren were then asked a battery of questions on the periodicity of care (with categories including weekdays, weekends, school holidays, throughout the year, or difficult to say). For each of the categories selected, grandparents were asked to report the frequency. For instance, those who reported looking after grandchildren at weekends were asked if that was mostly ‘every weekend’, ‘every other weekend’, or ‘less often’. Similarly, if a grandparent looked after grandchildren throughout the year or said that it was ‘difficult to say’, they were then asked if this had mostly been ‘4 to 7 days a week’, ‘2 to 3 days a week’, ‘1 day a week’, ‘up to a few days a month but not each week’ or ‘less often than once a month’. About 83% of grandparents selected only 1 periodicity of care, with the remaining combining between two (9%) and four options (4%). Given that most grandparents reported looking after grandchildren throughout the year, we constructed five categories which are broadly similar to the options available for this periodicity of grandparental childcare: (i) between 4 and 7 days a week; (ii) 2 to 3 days a week; (iii) 1 day a week; (iv) a few days a month; (v) less often than once a month or only on holidays. Respondents who selected other periodicities of care were categorised to their closest match: for instance, if someone reported care ‘every other weekend’ they would be classified as providing care ‘a few days a month’. If they selected ‘4 to 5 days’ on weekdays, they would be relabelled as providing grandchild care between 4 and 7 days a week. For those who selected 2 or more periodicities of care (17% of grandparents looking after grandchildren), we considered their highest frequency of grandchild care.

#### Grandchild care activities

Grandparents were asked to provide information on the activities undertaken with and for grandchildren. Grandparents who care for grandchildren were given a card listing a number of activities and were then asked which of them they did when they were looking after grandchildren. The following activities were included in our study: having grandchildren stay overnight; caring for them when sick; preparing meals for them; taking them to (or collecting them from) nursery or school; helping them with homework; playing with them and/or taking part in leisure activities. For each of the activities selected, grandparents were then asked if they were involved frequently, occasionally, or rarely. In our models, we grouped together ‘caring for’ (having them stay overnight or caring for them when ill) and ‘hands-on’ activities (cooking for and picking up grandchildren). For all indicators, we dichotomised tasks performed frequently versus those done less often or not selected at all.

#### Reasons for care

Finally, grandparents were asked to report the main reasons for looking after grandchildren. The following reasons were read out: to help them develop as people; it makes me feel engaged with young people; to help his/her/their parents go out to work; to give his/her/their parents a break; so his/her/their parents can go out in the evening; to help out financially; our family prefers family care; and it is difficult for me to refuse. Respondents could report all the reasons that applied to them, and less than 40 grandparents did not report any of them. In our analysis, we grouped together the following reasons: giving parents a break and allowing them to go out in the evening as both represent help for parents to take some time out from childcare responsibilities; financial help and help for working parents as they represent economic support for the family; and feeling engaged with young people and helping grandchildren to develop, as they represent emotional support. All reasons were then considered as binary indicators, with 1 indicating whether the respondent mentioned at least one of the reasons in each category and 0 otherwise.

### Main independent variables

As indicators of socio-economic circumstances, we included education and wealth. Whereas educational qualification may be gained in later life, in the cohorts under study highest qualification is a good indicator of educational experiences and outcomes in childhood and early adulthood. Educational level was recoded into three categories (low, middle, high) using the International Standard Classification of Education (http://www.uis.unesco.org/), where low education refers to no qualifications or less than O levels (or equivalent) and a high educational level is defined as having a university education or above. Wealth, unlike income, is less sensitive to labour market participation or occupation type and reflects one’s current socio-economic circumstances. Wealth—computed by the Institute for Fiscal Studies (Oldfield 2018) as the sum of savings, investments, and physical wealth minus debt—was categorised into quartiles.

### Potential confounders

A wide range of potential confounders related to demographic characteristics, socio-economic circumstances, and family characteristics were adjusted for in all multivariate analyses. The majority of these indicators are known to be associated with grandparental childcare provision (Bordone et al. 2016; Di Gessa et al. 2016, 2020; Fuller-Thomson and Minkler 2001; Hank and Buber 2009; Herlofson and Hagestad 2012; Igel and Szyklik 2011) as well as with the main independent variables under study (Agahi and Parker 2005; Scharf et al. 2005). In our analyses, we controlled for grandparents’ gender as grandmothers are more likely to provide care; and for their marital status (married/cohabiting vs unpartnered) as previous studies found that the presence of a partner is an important resource, as a partner can support and help to organise grandchild care (Di Gessa et al. 2020; Hank and Buber 2009). Given that participation in social activities and paid work is socio-economically patterned (Agahi and Parker 2005; Scharf et al. 2005) and has been shown to negatively affect regular provision of grandparental care (Arpino and Bordone 2017; Bordone et al. 2016; Di Gessa et al. 2016; Igel and Szyklik 2011), we also controlled for whether grandparents volunteered at least monthly (versus less often or not at all) and for their employment status, distinguishing between grandparents in paid work (part-time or full-time) and those retired or in ‘other occupations’ (including unemployed and homemakers). Moreover, given that younger grandparents are more likely to look after their grandchildren and do it more frequently (Fuller-Thomson and Minkler 2001; Hank and Buber 2009), we controlled for age (as a continuous variable) as well as a quadratic term to account for a nonlinear relationship. Also, prior studies suggest that grandparent health is an important factor affecting their ability to look after grandchildren (Di Gessa et al. 2016; Glaser et al. 2013). In our analysis, we then controlled for two indicators of health, that is depression measured by the validated Centre for Epidemiologic Studies Depression Scale (Beekman et al. 1997), with respondents reporting 4 or more depressive symptoms in the week prior to interview classified as having elevated depressive symptoms, and number of limitations defined as number of difficulties with basic as well as instrumental activities of daily living (ADL).

We also included several children’s and grandchild’s characteristics, as family structures have been associated with the provision of grandparental childcare (Aassve et al. 2012; Di Gessa et al. 2016; Herlofson and Hagestad 2012; Igel and Szyklik 2011; Thomese and Liefbroer 2013). Moreover, given links between socio-economic and family characteristics, differences in the experience of grandparental childcare by wealth and education may be accounted for by the fact that more socio-economically disadvantage groups tend to have smaller and more kin-based social networks, and to live in closer geographic proximity to their family members (Fors and Lennartsson 2008; Hank 2007; Litwin 2001). We included the total number of children and grandchildren grandparents had, as grandparents with more children/grandchildren may limit the amount of support they are able to provide to each (Di Gessa et al. 2016; Igel and Szyklik 2011). As previous studies indicate that geographical distance plays a substantial role in grandparents’ decision to help with grandchild care (Thomese and Liefbroer 2013), we controlled for time to travel to their nearest grandchild (living in the same household or less than 15 min away; between 15 and 30 min away; more than 30 min away). As only 53 grandparents (< 2% of the sample) were living with their grandchildren, it was not possible to consider these co-residential grandparents separately in our analysis. Finally, given that previous studies show that grandparents are more likely to look after their grandchildren when they are younger (Di Gessa et al. 2020; Fuller-Thomson and Minkler 2001) and that activities children undertake with their grandparents change with age (Dunifon et al. 2018), we considered the age of the youngest grandchild distinguishing between 0 to 2, 3 to 5, and 6 to 15 years. However, it is worth mentioning that the grandchildren’s characteristics in our sample (that is their distance and youngest age) do not necessarily refer to the grandchild grandparents were looking after.

### Statistical analysis

Preliminary analyses using an ordinal model for the frequency of grandparental childcare violated the proportional odds assumption (i.e. the relationship between each pair of outcome groups is the same). Therefore, we employed a multinomial logistic regression model to investigate the associations between grandparents’ socio-economic characteristics and frequency of grandchild care. To examine the socio-economic gradient in the experience of grandparental childcare, we ran logistic regressions for each of the activities and reasons for grandchild care described above. For all analyses, we present both unadjusted and fully adjusted results accounting for socio-demographic and health covariates and children’s and grandchildren’s characteristics. All analyses were performed using Stata 16.

## Results

### Descriptive findings

Table 1 shows the descriptive characteristics of the sample. About a quarter of grandparents who provided grandchild care were in paid work and had partaken in voluntary activities. Almost three out of four of these grandparents lived less than a half hour away from their closest grandchild, and about two-thirds reported having a youngest grandchild aged less than 6 years old.

**Table 1:**
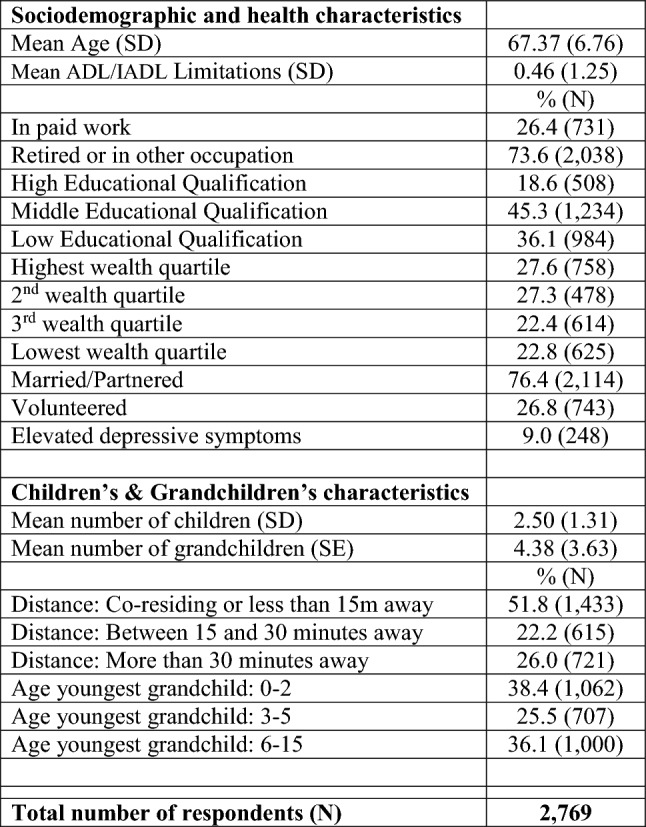
Grandparent sample characteristics

Source: ELSA, Wave 8 (2016–2017). Analyses are restricted to grandparents who reported grandparental childcare

Table 2 describes the frequency of grandchild care, activities undertaken and reasons for care. More than one quarter of grandparents (28%) reported 2 or more days per week of grandchild care, with eight percent looking after grandchildren almost daily. About one in six grandparents provided care to their grandchildren one day a week, whereas about one-third looked after grandchildren less often than a few days a month or during the school holidays. This table also shows that 41% of grandparents reported frequent engagement in leisure activities with grandchildren and 45% prepared meals for grandchildren or took them/collected them from school/nursery. As for reasons for grandchild care, three quarters of grandparents reported that they wanted to help parents (by giving them a break or allowing them to go out in the evening), almost 70% reported economic help and over half reported emotional help/support (help grandchildren develop as people or to feel engaged with young people). ‘Preference for family care’ and ‘It is difficult to refuse’ were mentioned less often as reasons for grandchild care.

**Table 2:**
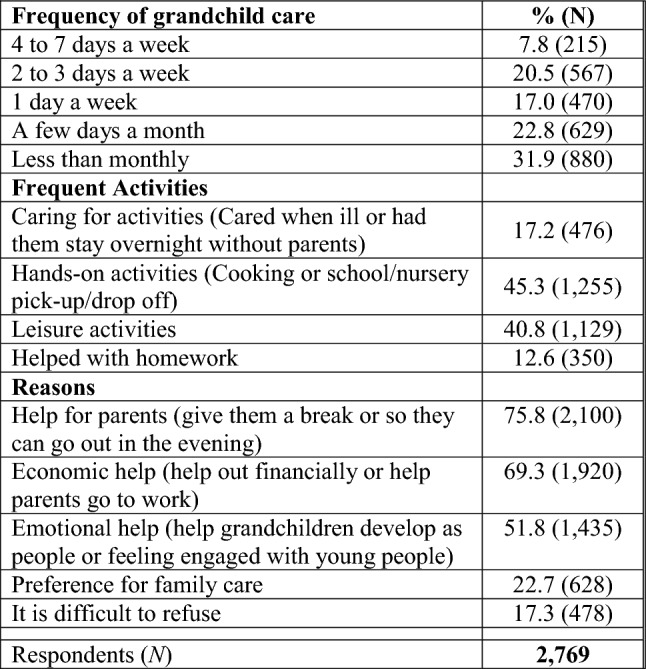
Frequency of grandchild care, activities, and reasons among grandparents who look after grandchildren

Source: ELSA, Wave 8 (2016–2017)

### Unadjusted and fully adjusted findings

Tables 3, 4 and 5 present unadjusted and fully adjusted results of the multinomial and logistic models which examine whether and to what extent grandparents’ socio-economic characteristics (education and wealth) are associated with the frequency of grandchild care provision (Table 3), the activities grandparents undertake frequently with and for their grandchildren (Table 4), and the reasons for care (Table 5). In the sections that follow, we only highlight statistically significant results with a p-value less than 0.05.

**Table 3:**
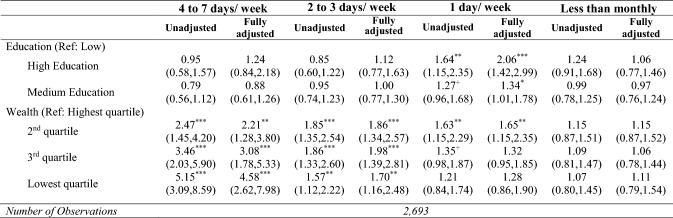
Associations between socio-economic characteristics and frequency of grandparental childcare

Results from un-adjusted and fully adjusted multinomial regression models—relative risk ratio (and 95% CIs) compared to ‘a few days a month’. *CI* confidence interval, *RRR* relative risk ratio. RRRs and 95% CIs obtained from un-adjusted and fully adjusted multinomial regression model (with ‘A few days a month’ as reference category). The fully adjusted model presented here (and available in full as Supplementary Table 1) adjusted for gender, age, age squared, marital status, employment status, volunteering, depression, functional limitations, number of children, number of grandchildren, distance to the closest grandchild, and age of the youngest grandchild. Source: ELSA, Wave 8. These analyses are restricted to grandparents who reported grandparental childcare

^+^*p* < 0.10

*p < 0.05

**p < 0.01

***p < 0.001

**Table 4:**
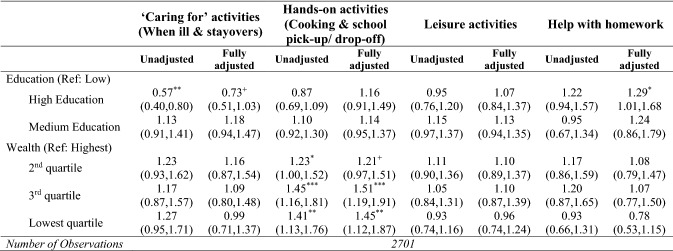
Associations between socio-economic characteristics and frequent childcare activities

Results from un-adjusted and fully adjusted logistic regression models—odds ratios (and 95% CIs)

CI = confidence interval. Odds ratios and 95% CIs obtained from un-adjusted and fully adjusted logistic regression models. The fully adjusted model presented here (and available in full as Supplementary Table 2) adjusted for gender, age, age squared, marital status, employment status, volunteering, depression, functional limitations, number of children, number of grandchildren, distance to the closest grandchild, and age of the youngest grandchild. Source: ELSA, Wave 8 (2016–2017). These analyses are restricted to grandparents who reported grandparental childcare

^ +^*p* < 0.10

*p < 0.05

**p < 0.01

***p < 0.001

**Table 5:**
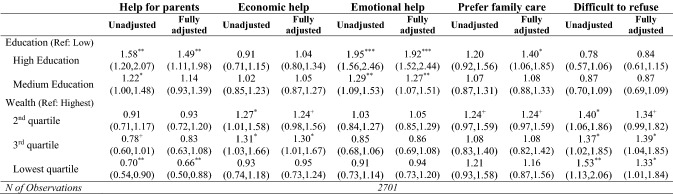
Associations between socio-economic characteristics and reasons for grandchild care

Results from un-adjusted and fully adjusted logistic regression models—odds ratios (and 95% CIs). *CI* confidence interval. Odds ratios and 95% CIs obtained from un-adjusted and fully adjusted logistic regression models. The fully adjusted model presented here (and available in full as Supplementary Table 3) adjusted for gender, age, age squared, marital status, employment status, volunteering, depression, functional limitations, number of children, number of grandchildren, distance to the closest grandchild, age of the youngest grandchild. Source: ELSA, Wave 8 (2016–2017). These analyses are restricted to grandparents who reported grandparental childcare

^+^*p* < 0.10

*p < 0.05

**p < 0.01

***p < 0.001

Table 3 presents the main effects for the unadjusted and fully adjusted relationship between socio-economic characteristics and frequency of grandchild care. Unadjusted results suggest that grandparents in the lowest wealth quartile group were more likely to provide grandchild care at least 2 days a week, with a clear wealth gradient that was particularly noticeable among grandparents who provided care almost daily. Unadjusted results also show that looking after grandchildren only once a week was higher for grandparents in the mid-wealth quartiles, whereas no associations were found between wealth and infrequent grandchild care. As for education, unadjusted results suggest that grandparents in the highest education group were more likely to provide grandchild care once a week than a few days a month. Even after adjusting for grandparents’ socio-demographic and health characteristics and for family structure, results showed similar associations between socio-economic characteristics and frequency of care.

Unadjusted associations between grandparents’ socio-economic characteristics and activities (Table 4) show that education was generally not associated with the activities grandparents undertook frequently with their grandchildren, except for highly educated grandparents who were less likely to undertake frequent ‘caring-for’ activities (overnights and caring for grandchildren when ill). Also, wealth was only associated with hands-on activities, with grandparents in the lowest wealth quartiles more likely than those in the highest wealth groups to cook for grandchildren and pick-up/drop-off them to/from school/nurseries. Once grandparents’ and their family’s characteristics were taken into account, results show that education was no longer associated with ‘caring-for’ activities, whereas a significant association was found with frequent help with homework: grandparents in the highest education group were more likely to undertake this activity than those in the lower educational attainment groups. As for wealth, fully adjusted results confirm that grandparents in the bottom two quartiles of the wealth distribution were more likely to prepare meals for their grandchildren and to collect/take them from/to school/nurseries.

Finally, Table 5 presents the main associations for the unadjusted and fully adjusted associations between grandparents’ socio-economic characteristics and reasons for grandchild care. Unadjusted results show that, compared to grandparents in the lowest education group, those in the medium and high education groups were more likely to report providing help to parents (giving them a break or allowing them to go out at night) as well as emotional help (helping grandchildren to develop as people and feeling engaged with young people). Unadjusted associations between wealth and reasons for care show that grandparents in the bottom wealth quartile were less likely to report help to parents but more likely to state that it was difficult for them to refuse compared to those in the highest wealth quartile. Also, grandparents in the mid-range wealth groups were more likely to report economic help (financial help and help for working parents) in comparison with their richer counterparts. In the unadjusted model, no associations were found between education, wealth, and preference for family care. When fully adjusted results were considered, associations remained broadly similar. However, once confounders were controlled for, highly educated grandparents were more likely than those with lower education levels to mention that they preferred family care.

## Discussion

Grandparents play a significant role in family life, particularly those looking after grandchildren: it is therefore important to understand whether and to what extent socio-economic inequalities in the provision of grandparental childcare exist. Using a suite of new questions on grandparenting that have been included in the most recent wave of the nationally representative ELSA, our aim was to understand the contribution of grandparental childcare to socio-economic inequalities among older people.

Overall, as suggested by the Informal Care Model, our results show that grandparental childcare is socio-economically patterned, with financially worse off grandparents more likely to provide grandchild care almost daily, and highly educated and better-off grandparents more likely to look after grandchildren less frequently (i.e. only once a week). Our study also found that highly educated grandparents were more likely to help with homework, whereas those in the lower wealth quartiles were more involved in hands-on activities, i.e. taking/collecting grandchildren to/from school/nursery and cooking for them. Finally, we found that grandparents in the mid/low wealth quartiles were more likely to care for economic reasons and because they found it difficult to refuse, whereas better-off grandparents were more likely to care to help their adult children (by giving them a break and enabling them to go out in the evening) and to provide emotional help (i.e. by helping grandchildren to develop and wanting to engage with young people). However, we also found that highly educated grandparents were more likely to declare a preference for family care. These findings mirror previous quantitative studies which focused on the middle generation (i.e. the grandchildren’s parents) and found that, once again, it was the mostly socio-economically disadvantaged parents who relied on grandparents as a primary source of childcare (Arpino et al. 2014; Kanji 2018; Laughlin 2013). They are also in line with qualitative studies (Airey et al. 2020; McGarrigle et al. 2018) which found that more highly educated grandparents chose relatively lower levels of involvement with grandchildren.

Our results show important variations in the associations between education, wealth, and grandparental childcare—also in line with the Informal Care Model. As this model would lead us to expect, wealth was mostly associated with frequency of grandchild care and with reasons for care being economic in nature (i.e. to help parents financially or to enable them to go to work). Also, poorer grandparents were more likely to provide hands-on help to their grandchildren (i.e. with cooking or picking up them up/dropping them off from school/nursery) and they were more likely to say that they provided grandchild care because it was difficult to refuse. This may be because poorer grandparents are less likely to have the financial resources with which to access and purchase alternatives to family care and support.

Finally, as expected, we also found an association between education and grandparental childcare. Education appears to be more of a proxy for grandparents’ control over both their level of involvement with grandchildren and their role within the family, with highly educated grandparents being more selective about how often they look after grandchildren, as well as what activities they undertake with them, and why (Conlon et al. 2014; King and Elder 1998). In line with findings based on the parents (or the middle-generation), highly educated grandparents were more likely to be involved in their grandchildren’s education (i.e. by helping with homework) (Bornstein and Putnick 2012; Guryan et al. 2008). Also, highly educated grandparents’ reasons for providing care highlight their willingness to help parents by giving them a break from childcare and aiding their grandchild(ren)’s development.

### Strengths and limitations

We investigated associations between grandparents’ socio-economic characteristics, frequency of grandchild care, activities undertaken for and with their grandchildren, and reasons for care. To our knowledge, this was the first study to investigate this issue among European grandparents using a large-scale nationally representative survey and to use a new module on grandparenting that also includes activities and reasons for care.

Our analyses, however, also have some limitations. First, as mentioned above, ELSA does not collect detailed information about the childcare provided to each grandchild, but rather asks a more generic question related to all grandchildren and ‘all the time’ spent looking after them. Although in our analyses we considered several grandchildren’s characteristics (such as the age of the youngest grandchild and where the nearest grandchild lives), we do not know if that is the grandchild grandparents had in mind when they answered questions about provision of grandchild care. Moreover, as suggested by the Informal Care Model, provision of informal care (and grandparental childcare in this case) is a ‘process in which individual, relational and contextual factors of both care recipient and caregiver are intertwined’ (Broese van Groenou and De Boer 2016:272). Therefore, to better understand the mechanisms that shape socio-economic differences in grandparental childcare, more information on the recipients of care, and on the micro-level (family, social network, and care in the community) and macro-level (caregiving resources and policies, norms, and preferences) contexts would be needed. Although the intergenerational decision-making process is generally related to the opportunities and resources of all three generations (Price et al. 2018), and parents’ marital and employment status are important determinants of the need for grandparents as providers of childcare (Di Gessa et al. 2016; Hank and Buber 2009; Igel and Szyklik 2011), ELSA does not collect any information on parents. Grandchild care may also reflect specific arrangements between parents and grandparents particularly when the former have no regular paid work or undertake ad hoc paid work making formal childcare arrangements difficult (Airey et al. 2020; Wheelock and Jones 2002). More generally, information on the socio-economic characteristics of the parents would shed some light on whether the results observed in this study are a reflection of the parent’s socio-economic characteristics, as adult children of grandparents from lower socio-economic groups are themselves from similar backgrounds (Fagereng et al. 2021; Roksa and Potter 2011) or an amplifier of socio-economic inequalities. Besides, as the information was not collected, we could not explore the quality of the intergenerational relationship between parents and grandparents which is also an important factor to consider when analysing intergenerational transfers. Similarly, we do not know which relatives other than grandparents are involved in grandchild care provision, although the use and combination of different informal childcare providers also reflects income and educational differences (Bryson et al. 2012). Moreover, we do not have information on the availability, access, and use of formal childcare (particularly for those aged 6 and younger) even though evidence suggests that more regular childcare provision is more prevalent where little formal childcare is available (Bordone et al. 2016; Di Gessa et al. 2016; Floridi 2020; Igel and Szyklik 2011). Given findings by Zamberletti et al. (2018), we acknowledge that the associations found in our study may vary across countries with different formal childcare settings, family norms, and employment policies; future studies using country-specific data sets are encouraged to explore this aspect.

## Conclusion

To conclude, our study shows that grandparents play an important role in family life, with most grandparents looking after their grandchildren at least weekly. However, grandparents who provide more frequent grandchild care are more likely to be socio-economically disadvantaged. Also, the role grandparents play in their grandchildren’s lives vary depending on their wealth and education, with higher socio-economic groups more likely to provide help for parents (i.e. to give them a break or allow them to go out for the evening) and emotional help to grandchildren (i.e. help them develop as people or wanting to feel engaged with young people). These findings suggest that the experience of grandparental childcare is not similar across grandparents of different socio-economic backgrounds, and their effects on inequalities in the distribution of family care and support responsibilities among older adults deserve policy attention. However, future research should aim to investigate how parents’ socio-economic and demographic characteristics affect and interact with socio-economic inequalities in grandparental childcare, as well as how activities, frequencies, and reasons for grandchild care interact with socio-economic status to affect grandparents’ health and well-being.

## Supplementary Information

Below is the link to the electronic supplementary material.

Supplementary file1 (PDF 185 kb)

## Data Availability

Researchers can download ELSA data from all waves, from the UK Data Service. For more information, please visit https://www.elsa-project.ac.uk/accessing-elsa-data.

## Code availability

All analyses were performed using Stata.
