# Supplementary material for: Is grandparental childcare socio-economically patterned? Evidence from the English longitudinal study of ageing
Source: Eur J Ageing. 2022 Jan 21;19(3):763–74. doi: 10.1007/s10433-021-00675-x (PMC9424417; doi:10.1007/s10433-021-00675-x)
Supplement: Supplementary file 1 — Supplementary file1 (PDF 185 kb) [file 10433_2021_675_MOESM1_ESM.pdf]

Supplementary Table 1. Associations between socio-economic, demographic, and health characteristics and frequency of grandparental childcare. Results from fully-adjusted multinomial regression models – Relative Risk Ratio (and 95% CIs)

	4 to 7 days/ week	2 to 3 days/ week	1 day/ week	Less than monthly
Education (Ref: Low)				
High Education	1.24 (0.84,2.18)	1.12 (0.77,1.63)	2.06*** (1.42,2.99)	1.06 (0.77,1.46)
Medium Education	0.88 (0.61,1.26)	1.00 (0.77,1.30)	1.34* (1.01,1.78)	0.97 (0.76,1.24)
Wealth (Ref: Highest quartile)				
2nd quartile	2.21** (1.28,3.80)	1.86*** (1.34,2.57)	1.65** (1.15,2.35)	1.15 (0.87,1.52)
3rd quartile	3.08*** (1.78,5.33)	1.98*** (1.39,2.81)	1.32 (0.95,1.85)	1.06 (0.78,1.44)
Lowest quartile	4.58*** (2.62,7.98)	1.70** (1.16,2.48)	1.28 (0.86,1.90)	1.11 (0.79,1.54)
Female	1.59* (1.11,2.28)	1.40** (1.08,1.80)	1.15 (0.88,1.49)	1.01 (0.81,1.27)
Age	1.05 (0.94,1.17)	1.14** (1.05,1.24)	1.12* (1.03,1.22)	0.99 (0.92,1.06)
Age squared	1.00 (1.00,1.00)	1.00*** (0.99,1.00)	1.00** (0.99,1.00)	1.00 (1.00,1.00)
Unpartnered (Ref: with partner)	1.29 (0.89,1.89)	0.94 (0.70,1.26)	1.06 (0.77,1.44)	0.86 (0.65,1.12)
In paid work (Ref: Not in paid work)	0.53** (0.34,0.86)	0.71* (0.53,0.98)	0.78 (0.56,1.09)	1.14 (0.86,1.53)
Volunteered (Ref: not volunteered)	0.86 (0.58,1.30)	0.79 (0.56,1.10)	1.13 (0.85,1.50)	0.96 (0.75,1.23)
Elevated depressive symptoms	0.81 (0.47,1.41)	0.74 (0.48,1.16)	1.11 (0.71,1.74)	0.84 (0.56,1.25)
ADL/IADL limitations	1.18** (1.04,1.34)	1.09 (0.97,1.22)	1.08 (0.96,1.21)	1.12* (1.01,1.24)
Number of children	0.87+ (0.74,1.01)	0.92 (0.83,1.02)	0.92 (0.82,1.04)	0.98 (0.89,1.07)
Number of grandchildren	0.95+ (0.90,1.01)	0.97 (0.93,1.01)	0.93** (0.89,0.98)	1.03+ (1.00,1.07)
Distance (Ref: ≤15m or cohabiting)				
Between 15 and 30m	0.30*** (0.19,0.48)	0.63** (0.48,0.85)	0.61** (0.45,0.83)	1.25 (0.95,1.65)
More than 30m	0.17*** (0.10,0.30)	0.20*** (0.14,0.29)	0.27*** (0.19,0.38)	2.36*** (1.82,3.06)
Age youngest grandchild (Ref: 0-2)				
3-5	1.95** (1.25,3.06)	1.00 (0.74,1.35)	1.26 (0.92,1.73)	1.38* (1.04,1.84)
6-15	1.91** (1.25,2.93)	0.61** (0.45,0.83)	0.78 (0.57,1.08)	1.75*** (1.34,2.28)
Number of Observations		*2,693*		

Notes. (I)ADL = (Instrumental) Activities of Daily Living; CI = confidence interval; RRR = relative risk ratio. RRRs and 95% CIs obtained from fully adjusted multinomial regression model (with monthly care as reference category). Source: ELSA, Wave 8. These analyses are restricted to grandparents who reported grandparental childcare. + p < 0.10, * p < 0.05, ** p < 0.01, *** p < 0.001.

Supplementary Table 2. Associations between socio-economic, demographic, and health characteristics and frequent childcare activities. Results from fully-adjusted logistic regression models – Odds Ratios (and 95% CIs)

	**Caring when ill & stayovers**	**Cooking & drop off/ pick up**	**Leisure activities**	**Help with homework**
Education (Ref: Low)				
High Education	0.73^{+} (0.51,1.03)	1.16 (0.91,1.49)	1.07 (0.84,1.37)	1.29* 1.01,1.68
Medium Education	1.18 (0.94,1.47)	1.14 (0.95,1.37)	1.13 (0.94,1.35)	1.24 (0.86,1.79)
Wealth (Ref: Highest quartile)				
2nd quartile	1.16 (0.87,1.54)	1.21^{+} (0.97,1.51)	1.10 (0.89,1.37)	1.08 (0.79,1.47)
3rd quartile	1.09 (0.80,1.48)	1.51*** (1.19,1.91)	1.10 (0.87,1.39)	1.07 (0.77,1.50)
Lowest quartile	0.99 (0.71,1.37)	1.45** (1.12,1.87)	0.96 (0.74,1.24)	0.78 (0.53,1.15)
Female	1.32* (1.06,1.66)	2.01*** (1.69,2.38)	1.30** (1.10,1.55)	1.45** (1.13,1.88)
Age	1.00 (0.93,1.07)	1.09** (1.03,1.15)	1.05^{+} (0.99,1.11)	1.05 (0.97,1.15)
Age squared	1.00 (1.00,1.00)	1.00*** (1.00,1.00)	1.00** (1.00,1.00)	1.00* (1.00,1.00)
Unpartnered (Ref: with partner)	1.04 (0.81,1.33)	1.07 (0.87,1.30)	1.05 (0.86,1.29)	1.24 (0.94,1.64)
In paid work (Ref: Not in paid work)	1.27^{+} (0.97,1.67)	0.70** (0.56,0.88)	0.75** (0.60,0.93)	0.99 (0.73,1.35)
Volunteered (Ref: not volunteered)	0.92 (0.71,1.18)	1.01 (0.83,1.22)	1.00 (0.83,1.21)	1.02 (0.77,1.34)
Elevated depressive symptoms	1.31 (0.93,1.86)	0.93 (0.69,1.25)	0.65** (0.48,0.88)	0.85 (0.54,1.32)
IADL/ADL limitations	1.02 (0.94,1.11)	0.92* (0.86,0.99)	0.98 (0.91,1.05)	0.98 (0.88,1.08)
Number of children	0.96 (0.88,1.06)	0.94 (0.88,1.02)	1.02 (0.95,1.09)	1.02 (0.92,1.14)
Number of grandchildren	1.01 (0.97,1.04)	0.98 (0.95,1.01)	0.95*** (0.92,0.98)	1.02 (0.98,1.05)
Distance (Ref: ≤15m or cohabiting)				
Between 15 and 30m	0.78^{+} (0.61,1.01)	0.64*** (0.52,0.78)	0.79* (0.64,0.96)	0.81 (0.61,1.07)
More than 30m	0.38*** (0.28,0.51)	0.29*** (0.23,0.36)	0.39*** (0.32,0.48)	0.35*** (0.25,0.49)
Age youngest grandchild (Ref: 0-2)				
3-5	0.90 (0.69,1.18)	1.16 (0.94,1.43)	0.97 (0.79,1.18)	1.69*** (1.26,2.27)
6-15	1.08 (0.84,1.40)	0.85 (0.70,1.04)	0.53*** (0.43,0.64)	1.40* (1.04,1.89)
Number of Observations		*2701*		

Notes. (I)ADL = (Instrumental) Activities of Daily Living; CI = confidence interval. Odds ratios and 95% CIs obtained from logistic regression models. Source: ELSA, Wave 8 (2016-2017). These analyses are restricted to grandparents who reported grandparental childcare. + $p < 0.10$, * $p < 0.05$, ** $p < 0.01$, *** $p < 0.001$.

Supplementary Table 3. Associations between socio-economic, demographic, and health characteristics and reasons for grandchild care. Results from fully-adjusted logistic regression models – Odds Ratios (and 95% CIs)

	**Help for parents**	**Economic help**	**Emotional help**	**Prefer family care**	**Difficult to refuse**
Education (Ref: Low)					
High	1.49** (1.11,1.98)	1.04 (0.80,1.34)	1.92*** (1.52,2.44)	1.40* (1.06,1.85)	0.84 (0.61,1.15)
Medium	1.14 (0.93,1.39)	1.05 (0.87,1.27)	1.27** (1.07,1.51)	1.08 (0.88,1.33)	0.87 (0.69,1.09)
Wealth (Ref: Highest quartile)					
2nd quartile	0.93 (0.72,1.20)	1.24+ (0.98,1.56)	1.05 (0.85,1.29)	1.24+ (0.97,1.59)	1.34+ 0.99,1.82
3rd quartile	0.83 (0.63,1.08)	1.30* (1.01,1.67)	0.86 (0.69,1.08)	1.08 (0.82,1.42)	1.39* 1.04,1.85
Lowest quartile	0.66** (0.50,0.88)	0.95 (0.73,1.24)	0.94 (0.73,1.20)	1.16 (0.87,1.56)	1.33* 1.01,1.84
Female	0.96 (0.79,1.16)	1.01 (0.85,1.21)	0.98 (0.83,1.15)	1.45*** (1.19,1.77)	0.97 (0.78,1.20)
Age	0.99 (0.93,1.05)	1.13*** (1.07,1.19)	1.04 (0.99,1.10)	1.05 (0.99,1.12)	1.01 (0.94,1.08)
Age squared	1.00 (1.00,1.00)	1.00*** (1.00,1.00)	1.00* (1.00,1.00)	1.00* (1.00,1.00)	1.00 (1.00,1.00)
Unpartnered (Ref: with partner)	0.93 (0.75,1.16)	1.21+ (0.98,1.50)	1.01 (0.84,1.23)	0.96 (0.77,1.21)	0.90 (0.70,1.16)
In paid work (Ref: Not in paid work)	1.03 (0.80,1.33)	0.91 (0.72,1.15)	1.11 (0.90,1.37)	0.89 (0.70,1.15)	1.13 (0.86,1.47)
Volunteered (Ref: not volunteered)	0.90 (0.73,1.11)	1.16 (0.95,1.42)	1.29** (1.07,1.54)	1.15 (0.92,1.42)	1.14 (0.90,1.46)
Elevated depressive symptoms	0.73* (0.53,1.00)	0.81 (0.60,1.10)	1.19 (0.89,1.58)	0.94 (0.67,1.31)	1.46* (1.03,2.05)
IADL/ADL limitations	1.00 (0.93,1.08)	0.98 (0.91,1.05)	0.99 (0.93,1.06)	1.04 (0.96,1.12)	1.03 (0.95,1.12)
Number of children	1.05 (0.97,1.15)	0.98 (0.91,1.06)	0.98 (0.91,1.05)	0.95 (0.87,1.03)	0.97 (0.88,1.06)
Number of grandchildren	0.99 (0.96,1.02)	0.99 (0.96,1.02)	1.00 (0.97,1.02)	1.01 (0.98,1.05)	1.01 (0.97,1.04)
Distance (Ref: ≤15m or cohabiting)					
Between 15 and 30	1.07 (0.85,1.34)	0.72** (0.58,0.89)	1.01 (0.83,1.23)	0.83 (0.66,1.05)	0.76* (0.59,0.99)
More than 30m	1.05 (0.83,1.31)	0.43*** (0.35,0.52)	0.97 (0.80,1.17)	0.54*** (0.43,0.69)	0.58*** (0.44,0.76)
Age youngest grandchild (Ref: 0-2)					
3-5	0.79* (0.62,1.00)	1.19 (0.95,1.49)	1.30** (1.07,1.59)	1.25+ (1.00,1.58)	1.15 (0.90,1.49)
6-15	0.63*** (0.51,0.80)	0.87 (0.70,1.07)	1.16 (0.96,1.41)	0.93 (0.74,1.17)	0.83 (0.64,1.07)
Number of Observations			*2701*		

Notes. (I)ADL = (Instrumental) Activities of Daily Living; CI = confidence interval. Odds ratios and 95% CIs obtained from logistic regression models. Source: ELSA, Wave 8 (2016-2017). These analyses are restricted to grandparents who reported grandparental childcare. + p < 0.10, * p < 0.05, ** p < 0.01, *** p < 0.001
